# Impact of *Romanov* breed lamb gender on carcass traits and meat quality parameters including biogenic amines and malondialdehyde changes during storage

**DOI:** 10.1002/fsn3.2793

**Published:** 2022-03-11

**Authors:** Dovile Klupsaite, Vilija Buckiuniene, Saulius Bliznikas, Sonata Sidlauskiene, Agila Dauksiene, Jolita Klementaviciute, Andrius Jurkevicius, Gintare Zaborskiene, Elena Bartkiene

**Affiliations:** ^1^ Institute of Animal Rearing Technology Lithuanian University of Health Sciences Kaunas Lithuania; ^2^ Department of Food Safety and Quality Faculty of Veterinary Medicine Lithuanian University of Health Sciences Kaunas Lithuania

**Keywords:** biogenic amines, carcass trait, fatty acid profile, meat quality, *Romanov* lamb

## Abstract

The present study aims to evaluate the effect of *Romanov* breed lamb gender on carcass traits and meat quality parameters, as well as on the formation of biogenic amines (BAs) and malondialdehyde during meat storage. Obtained results revealed that lamb gender had a significant influence on sternum/breastbone, ribs, right shoulder, and bones of the back leg. Significantly higher lightness (by 3%) was found for male meat; however, higher redness of female meat was observed (by 7.7%). In all cases, a lower pH was obtained for female meat. Significantly higher cooking loss (by 38%) was found for male meat. However, gender was not a significant factor in lamb meat proximate composition, or for BAs and cholesterol content. The gender of animals had a significant influence on 10‐heptadecenoic (C17:1), linoleic (C18:2n – 6), total polyunsaturated FA, and total *trans* isomers content in meat. A significantly higher concentration of malondialdehyde was found in female lamb meat (by 43.4% and 56.8% in fresh and after 3 months of storage at −18°C, respectively) compared to males. Finally, the obtained results supplement the scarce database about the characteristics of *Romanov* breed meat of different gender and this is beneficial for lamb breeders and meat industry in order to obtain a better quality production.

## Graphical abstract

Study aimed to examine the influence of gender on carcass traits and meat quality of Romanov breed lambs. Lamb gender had a significant influence on several carcass traits, 10‐heptadecenoic, linoleic, and total polyunsaturated fatty acids profile. Male meat had a significantly higher lightness, lower redness, and higher values of pH and cooking loss. A significantly higher malondialdehyde content was found in female lamb meat. Gender was not a significant factor in lamb meat proximate composition, cholesterol concentration, or the content of most biogenic amines.

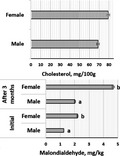

## 1 INTRODUCTION

Global meat consumption is increasing with increased human population and economic growth (González et al., 2020). Nowadays, consumers are looking for meat, which is safe, of high quality, and nutritious (Aboah & Lees, 2020). Due to this demand, the present trend in meat production is to focus not only on quantity but also on the quality of red meat (Teixeira & Rodrigues, 2019).

Lamb farming is an important livestock activity worldwide, being a good source of meat and wool. Lamb meat is an excellent source of animal protein, as well as important minerals (iron and zinc) (Karabagias, 2018). The *Romanov* is one of the most productive purebred sheep breeds, originated in Russia (Dvalishvili et al., 2015). The *Romanov* breed is characterized by high productive and reproductive performance with a high number of juveniles, as a *Romanov* ewe can produce seven live and healthy lambs in a litter (Zapasnikiene & Nainiene, 2012).

In general, meat quality is influenced by animal age, gender, physiological state, post‐mortem biochemistry, carcass composition, and feed contribution to flavor, protein, and fat levels, as well as the effect of genetics on tissues and metabolism, pre‐ and post‐slaughter handling, storage, etc. (Armstrong et al., 2018). Nowadays, there is growing interest in manipulating the carcass quality and fatty acid (FA) composition of livestock meat in order to produce meat with higher content of polyunsaturated FA (*n‐3*) and better consumer acceptance (Vahmani et al., 2020). A number of strategies have been used to supply sheep meat according to this new consumer demand (Hopkins & Mortimer, 2014). Some animal production strategies affect the chemical and physical quality of the meat, and usually, to obtain desirable production rates and carcasses with different characteristics, animals of different gender are selected; breed also has a large effect on carcass morphology (Hopkins & Mortimer, 2014).

Quite a range of parameters (e.g., pH, color, fat content, carcass, etc.) characterizes the quality of lamb meat and most of them are widely explored (Corazzin et al., 2019). However, other not so often mentioned compounds or processes, such as formation of biogenic amines (BAs) or malondialdehyde (MDA), could be also relevant for meat quality and consumer health. BAs have been reported in a variety of foods as the result of the microbial decarboxylation of amino acids (Papageorgiou et al., 2018). These compounds mostly participate as neurotransmitters in human body but when a high content of BAs is consumed with food, this leads to the intoxication of the organism (Ruiz‐Capillas & Herrero, 2019). The most common symptoms include headache, abdominal cramps, nausea, diarrhea, breathing difficulties, low or highly increased blood pressure, and rash (Wójcik et al., 2020). Increased content of BAs could be related with the lack of hygiene and usually reflects the freshness of the food (Ruiz‐Capillas & Herrero, 2019). For this reason, the control of BAs in foods is very important. Another important issue for meat quality is lipid peroxidation, which leads to undesirable changes in meat color, flavor, odor, texture, and even nutritional value during storage (Cimmino et al., 2018). Besides, lipid peroxidation, which is initiated in the subcellular membranes of the unsaturated fatty acid (UFA) fraction, is a major cause of the deterioration and reduced shelf life of meat products (Yagoubi et al., 2018). Changes that occur in the muscle during the post‐mortem period may create an unbalanced proportion between antioxidant and pro‐oxidant capability, increasing the risk of oxidative damage (Sabow et al., 2016). MDA is one of the lipid peroxidation products and widely known as the marker of this process (Ma et al., 2020). It was reported that MDA elicits a great concern to human health (Douny et al., 2015). Therefore, the composition and content of lipid fraction is a very important indicator of meat quality (Corazzin et al., 2019).

Several reports have shown that male lambs have greater daily body gain, lower carcass fat (Bas et al., 2007), and a better feed conversion ratio and daily weight gain (Kashani & Bahari, 2017) than females. On the contrary, there is conflicting evidence about the influence of gender on meat physical parameters. On the contrary, there is conflicting evidence about the influence of gender on meat physical parameters and FA composition of muscle lipids. Tejeda et al. (2008) reported that color, pH, moisture, and intramuscular fat were not influenced by lamb gender, while total polyunsaturated fatty acid (PUFA) content was higher in females than in males. Yarali et al. (2014) found that female Kıvırcık lambs had a lower PUFA content and higher cooking loss and water loss that males; however, a significant difference in meat shear force between genders was not observed. The study of Facciolongo et al. (2015) showed that meat of the *Gentile di Puglia* males had higher values of final pH, brightness, and yellow index, and a lower PUFA n‐6:n‐3 ratio in comparison with females. In this respect, there is still a need for the more detailed studies in this field. Therefore, the present study aims to evaluate the effect of *Romanov* breed lamb gender on carcass traits and meat quality parameters, as well as on the formation of biogenic amines and malondialdehyde during meat storage.

## 2 MATERIALS AND METHODS

### 2.1 Animals used in the study

The animals were cared for in accordance with the Lithuanian State Food and Veterinary Service requirements (“Requirements for the Keeping, Maintenance and Use of Animals Intended for Science and Education Purposes,” approved by the order of the Lithuanian Director of the State Food and Veterinary Service, 31/10/2012, No. B1‐866). Lambs of Romanov breed were reared at the commercial sheep farm in Kaunas district (Lithuania). Lambs were born by natural way during March and April and reared on the same farm where they were born, until slaughter. All animals were kept with their mothers on natural pasture under the same rearing conditions, and they were fed ad libitum with ewe's milk and grasses. Water was given freely to all animals. At 6 months of age, the lambs were divided into two groups according to their gender (group I – male lambs, and group II – female lambs) and transported to a commercial slaughterhouse. A total of 34 lambs (17 males and 17 females) were randomly selected for this study.

### 2.2 Evaluation of lamb carcass traits

After weighing, the animals were electrically stunned and slaughtered according to standard commercial procedures. The non‐carcass components, which included the head, skin, feet, kidneys, internal fat, testicles, tail, gastrointestinal tract, lungs plus trachea, heart, liver, and spleen, were removed.

Carcasses were weighed immediately after dressing the animals to obtain hot carcass weight (HCW). The carcasses were then chilled at 4°C for a 24‐h period and reweighed to obtain cold carcass weight (CCW). Carcass length, corresponding to the distance from the base of the tail to the base of the neck, was measured with a flexible measuring tape (Wintape Measuring Tape Co., Guangdong, China). Carcasses were carefully split longitudinally to obtain left and right halves. The carcasses were jointed into eight standard commercial joints namely neck, shoulder, ribs, legs, loins, and pelvis. The total weight of each joint was recorded. Each region was dissected into muscle, subcutaneous fat, intramuscular fat and bones, and weighed. The remainder, such as major blood vessels, ligaments, and tendons, were removed (Shija et al., 2013). After the evaluation of carcass traits, the samples of *Gluteus medius* muscle were transported (at 4°C) in a portable chiller to the Laboratory of Animal and Aquaculture Productivity and Production Quality of the Lithuanian University of Health Sciences (Kaunas, Lithuania).

### 2.3 Evaluation of lamb meat physicochemical parameters

The *Gluteus medius* muscle was used for meat chemical analysis (protein, fat, ash, and dry matter), determination of cholesterol, biogenic amines, malondialdehyde, and fatty acids profile, as well as for the assessment of meat quality traits (pH, color, water holding capacity, cooking loss, and shear force). Until analysis of meat quality, samples were vacuum packed and kept at 4°C in the refrigerator. The muscle pH was measured at 24 and 48 h post‐mortem. The analysis of meat color, water holding capacity, cooking loss, shear force, fatty acids, biogenic amines, and malondialdehyde was done at 48 h post‐mortem. The rest of samples were vacuum packed and frozen at –18°C until further analysis.

The analysis of meat physicochemical parameters was carried out as described by Rozanski et al. (2017) and AOAC (2019). Measurement of muscle pH was done with a pH meter (model Inolab 3, Hanna Instruments, Italy), calibrated to pH 4.0 and 7.0. Meat color (*L* ^∗^, *a* ^∗^, and *b* ^∗^ *)* was measured after a blooming period of 30–40 min and remeasured in the middle of the day, using a Minolta Chroma Meter colorimeter (CR‐400, Minolta Camera, Osaka, Japan) with a closed cone, set on the *L* ^∗^, *a* ^∗^, and *b* ^∗^ system. The chromameter was calibrated with a white tile (*Y* = 92.8, *x* = 0.3160, *y* = 0.3323) using Illuminant D‐65, with 2° standard observer and an aperture size of 8 mm. The determination of water holding capacity (WHC), drip loss (DL), cooking loss (CL), and shear force (SF) was performed as described in Klupsaite et al. (2020). WHC was determined using filter paper press method. Briefly, sample (2 g) was placed on a filter paper (Whatman filter paper 41/ashless), compressed between two plexiglass sheets, and received a pressure exerted by a weight of 1 kg for 10 min. DL was measured as the weight loss during suspension of a standardized (40–50 g and approximately 30 × 60 × 25 mm) muscle sample (in an airtight container over 24 h at 4°C). The CL corresponded to the weight difference between the samples (in a plastic container) before and after cooking in a water bath (internal temperature of 70°C for 30 min). For the evaluation of SF, three cylindrical samples with a diameter of 1.27 cm were removed from each sample that was used to determine CL. SF was measured using TA‐XT Plus texturometer (TA.XT plus Texture Analyzer, Stable Microsystems Ltd., Surrey, England) coupled to a Warner‐Bratzler device. The dry matter content of muscles was determined after oven drying the samples at 105°C for 24 h. Ash was determined by burning meat samples in a muffle furnace at 550°C for 4 h. Intramuscular fat content was determined using a Soxhlet SE 416 Columbus macro automatic system for fat extraction (Gerhardt, Germany). The protein content in meat samples was analyzed by the Kjeldahl method (ISO 937:1974).

### 2.4 Evaluation of biogenic amine content in lamb meat

The analysis of BAs content was performed at 48 h post‐mortem. After that, these samples were vacuum packed, stored at −18°C in freezer for 3 months, and used for further determination of BAs. The assessment of Bas was performed according to the method of Ben‐Gigirey et al. (1999) with some modifications. Following BAs were analyzed: tryptamine (TRY), phenylethylamine (PHE), cadaverine (CAD), putrescine (PUT), histamine (HIS), tyramine (TYR), spermine (SPER), and spermidine (SPRMD). Sodium hydroxide, perchloric acid, dansyl chloride, ammonium hydroxide, sodium bicarbonate, acetonitrile (HPLC grade), and ammonium acetate were obtained from Sigma‐Aldrich (St. Louis, Missouri, USA). Tryptamine hydrochloride, 2‐phenylethylamine hydrochloride, 1,4‐diaminobutane dihydrochloride, cadaverine dihydrochloride, histamine dihydrochloride, tyramine hydrochloride, spermidine phosphate salt hexahydrate, and 1,7‐diaminoheptane were obtained from Sigma‐Aldrich (St. Louis, Missouri, USA). Spermine diphosphate hexahydrate was from TCI Europe (Tokyo, Japan). All reagents were of analytical grade.

The standard BA solutions were prepared by dissolving known amounts of each BAs (including internal standard – 1.7‐diamino‐heptane) in 20 ml of deionized water. Briefly, the extraction of Bas in samples (5 g) was done by using 0.4 mol/L perchloric acid. The derivatization of sample extracts and standards was performed using a dansyl chloride solution in acetonitrile (10 mg/ml) as a reagent. The Varian ProStar HPLC system (Varian Corp., Palo Alto, California, USA) was made up of the following: two ProStar 210 pumps, a ProStar 410 autosampler, a ProStar 325 UV/VIS Detector, and Galaxy software (Agilent, Santa Clara, California, USA) for data processing. For the separation of amines, a Discovery^®^ HS C18 column (150 × 4.6 mm, 5 µm; SupelcoTM Analytical, Bellefonte, Pennsylvania, USA) was used. The eluents were ammonium acetate (A) and acetonitrile (B) and the elution program consisted of a gradient system with a 0.8 ml/min flow rate. The detection wavelength was set to 254 nm, the oven temperature was 40°C and samples were injected in 20 μl aliquots. The target compounds were identified based on their retention times in comparison to their corresponding standards. The results were expressed as milligrams per kilogram of sample. The extraction of BAs with 0.4 M perchloric acid was repeated twice. All analytical determinations were performed at least in triplicate.

### 2.5 Analysis of fatty acid content in lamb meat

Extraction of lipids for FA analysis was performed with chloroform/methanol (2:1 v/v) as described by Pérez‐Palacios et al. (2012). Fatty acid methyl esters (FAME) were prepared by esterification with 2 mol/L of KOH in methanol and shaking using laboratory shaker for 1 hr, upper layer was filtered using 0.22 µm membrane syringe filter and used for the analysis. Fatty acid composition was determined using gas chromatograph GC‐2010 Plus (Shimadzu corp.) equipped with Mass Spectrometer GCMS‐QP2010 (Shimadzu corp.). Separation was carried out on a Rxi‐5ms column (30 m length, 0.25 mmID, and 0.25μm *df* (Restek). Mass spectrometer was operated at full scan mode. Analyte was injected in split mode at 1:60 split ratio. Oven temperature programming started at 40°C, it was raised 8°C/min to 220°C, held for 1 min at 220°C, increased again at 20°C/min to 270°C, and held for the last 10 min. Injector temperature was 240°C, interface −270°C, and ion source 220°C. The carrier gas was helium at a flow rate of 0.91 ml/min. Fatty acid methyl esters (FAME) concentration was determined using calibration curve and results were expressed as percentage of total FAME concentration in sample. The calibration curve was prepared using standard Supelco 37 Component FAME Mix (Merck & Co., Inc.). The extraction of lipids was performed once and all analytical determinations were performed at least in triplicate.

### 2.6 Evaluation of cholesterol and malondialdehyde content in lamb meat

The analysis of cholesterol was performed according to the method of Polak et al. (2008) with some modifications. Lamb meat sample (2.00 ± 0.01 g) and 10 ml of saturated KOH were mixed and heated for 60 min at 60°C. The sample tube was cooled and 10 ml of hexane was added. The distilled water (3 ml) was poured into top layer of liquid and then the tube was shaken for 10 times. The sample was then centrifuged at 1000 *g* for 2 min (Sigma 2–5, Germany). An aliquot of the hexane extract (1 ml) was transferred to vial, dried with a nitrogen flush, and dissolved in 2 ml of mobile phase. The HPLC instrumentation was used the same as mentioned for BAs analysis. The mobile phase comprised a mix of 2‐propanol and acetonitrile (45:55) at a flow rate of 1.0 ml/min. The chromatograms were processed at a wavelength of 210 nm. The results were expressed as milligrams per 100 grams of sample. The extraction was performed once and all analytical determinations were performed at least in triplicate.

The analysis of MDA was performed at 48 hr post‐mortem. After that, these samples were vacuum packed, stored at −18°C in freezer for 3 months, and used for further determination of MDA. MDA was analyzed according to method described by Mendes et al. (2009). Trichloroacetic acid (≥99.5% purity), acetonitrile (HPLC grade), KH_2_PO_4_, and methanol (HPLC grade) were purchased from Sigma‐Aldrich (Saint Louis, Missouri, USA). 2‐Thiobarbituric acid (TBA) (≥99% purity) was obtained from Merck (Darmstadt, Deutschland).

Five gram of sample and 30 ml of 7.5% trichloracetic acid solution were mixed and homogenized with homogenizer (IKA T18, IKA^®^‐Werke GmbH & Co. KG, Staufen, Germany) for 1 min and filtered through filter paper (Whatman #1). Filtrate was again centrifuged for 15 min at 3000 g (Sigma 2‐5, Sigma Laborzentrifugen GmbH, Osterode am Harz, Germany). To derivatize MDA, 1.5 ml of TBA solution (40 mM) was added to supernatant (extract). The mixture was shaken and incubated in a heating oven (97°C) for 60 min. After cooling the sample (for 25 min at −18°C), 3 ml of methanol was added. The solution was shaken vigorously in a vortex and filtered through a PTFE syringe filter into a vial for HPLC. The sample was analyzed using the same HPLC system as for BAs analysis with some modifications: Varian ProStar 363 fluorescence detector (ʎ_excitation_ = 525 nm and ʎ_emission_ = 560 nm) (Varian Corp., Palo Alto, California, USA) and Phenomenex Gemini C18 column (5 μm, 250 × 4.6 mm) (Torrance, California, USA). The mobile phase comprised a mix of 50 mM KH_2_PO_4_, methanol, and acetonitrile (72:17:11); the elution program consisted of an isocratic system with a 1.0 ml/min flow rate. Results were expressed as milligrams in kilogram of sample. The extraction was performed once and all analytical determinations were performed at least in triplicate.

### 2.7 Statistical analysis

All measurements were carried out in triplicate. Statistical analysis was performed using the descriptive statistics and univariate analysis of variance (ANOVA) (statistical program R 3.2.1; R Core Team, 2015) to determine the significance effect of lamb gender on the carcass traits and meat quality parameters. The mentioned parameters were set as dependent variables and gender as an independent variable. The assumption of normality and homogeneity of variance of data were assessed by the Shapiro–Wilk and Levene's tests, respectively. Results were considered statistically significantly different at *p* ≤ .05.

## 3 RESULTS AND DISCUSSION

### 3.1 Influence of lamb gender on carcass traits

The effect of lamb gender on carcass traits is shown in Table 1. According to the results obtained, lamb gender has a significant influence on the sternum/breastbone (male sternum/breast was 13.9% heavier), ribs (male ribs were 12.6% heavier), right shoulder (male right shoulder was 15.2% heavier), and bones (left and right) of the rear foot (male bones were 10.3% and 20.5% heavier, respectively). Carcass weight, yield, and fat measurements are parameters of great importance to producers; weight and fat measurement can also determine the price paid for lamb carcasses (Gardner et al., 2015). Many studies have been conducted in relation to the evaluation of lamb carcass characteristics, and it is important to estimate the impacts on the quantity and quality of muscles in a carcass (De Brito et al., 2016). Gender can influence carcass composition because female lambs have a greater proportion of fatty tissue than males. However, the effect on meat quality traits is less clear (Santos et al., 2015). Ram lambs are favored in lamb production systems for their faster growth rates compared to ewe, castrated, and cryptorchid lambs (Kenyon & Schreurs, 2017). The meat from ram lambs has been associated with poorer tenderness and color characteristics, in comparison with meat from ewe lambs (Stempa et al., 2018). An influence of animal gender is evident in the regulation of adiposity and muscularity, and these differences have been attributed to gender steroid hormones because testosterone promotes an increase in body mass (de Araújo et al., 2017). From an economic point of view, the carcass is the most valuable part of the animal, and at certain weights it can be largely breed dependent (Mateo et al., 2018).

**TABLE 1:** Effect of *Romanov* lamb gender on carcass traits

Parameter	Male lamb	Female lamb
Hot carcass weight, kg	22.18 ± 0.97	21.74 ± 0.76
Cold carcass weight, kg	22.07 ± 0.89	20.65 ± 0.74
Carcass length, cm	51.82 ± 2.13	49.82 ± 2.63
Carcass yield, %	47.82 ± 1.98	48.21 ± 1.86
Sternum/breastbone, kg	0.72 ± 0.02^b^	0.62 ± 0.04^a^
Neck weight, kg	2.82 ± 0.24	2.48 ± 0.08
Ribs, kg	6.27 ± 0.31^b^	5.48 ± 0.20^a^
Front leg (left), kg	2.38 ± 0.14	2.34 ± 0.11
Right shoulder (muscle), kg	1.91 ± 0.13^b^	1.62 ± 0.11^a^
Bone of shoulder, kg	0.45 ± 0.02	0.43 ± 0.02
Front leg (right), kg	2.28 ± 0.11	2.23 ± 0.11
Left shoulder (muscle), kg	1.89 ± 0.12	1.83 ± 0.10
Bone of shoulder, kg	0.45 ± 0.02	0.42 ± 0.02
Back leg (left), kg	2.34 ± 0.08	2.30 ± 0.09
Bone of rear foot, kg	0.39 ± 0.01^b^	0.35 ± 0.01^a^
Ham, kg	1.97 ± 0.10	1.89 ± 0.10
Back leg, kg	2.33 ± 0.10	2.25 ± 0.08
Bone of back leg, kg	0.39 ± 0.01^b^	0.31 ± 0.01^a^
Ham, kg	1.95 ± 0.10	1.99 ± 0.10
Loins, kg	1.49 ± 0.06	1.52 ± 0.11
Loins and pelvis together, kg	3.20 ± 0.14	3.36 ± 0.16

Note

Data expressed as means (*n* = 17) ± standard error.

^a,b^Mean values with different letters along the row are significantly different (*p* ≤ .05).

### 3.2 Differences in meat quality parameters and chemical composition between lambs of different gender

Male and female lamb meat technological parameters are shown in Table 2. In all cases, lamb gender was a significant factor influencing lamb meat color coordinates (L* *p* = .045; a* *p* = .023; b* *p* = .006). Higher lightness (L* coordinates higher by 3.0%) of male meat was found. Opposite to the lightness, higher redness and yellowness coordinates of female meat were obtained (7.7% and 32.0% higher, respectively). The color coordinates of lamb meat can be related to animals’ diet, production system, slaughter weight, breed, gender, and muscle type (Kuchtík et al., 2012). In general, consumers rate color as the most important quality trait of fresh meat (della Malva et al., 2016). Some confusion exists in the literature regarding the effect of gender on lamb meat color, with some authors reporting significantly higher lightness values in lamb males, and others no differences between genders (Santos et al., 2015). The L* coordinate of meat depends on the pigment content, especially that of hematin, myoglobin, and their forms. Chromophores, such as myoglobin and hemoglobin, absorb visible light by increasing light penetration and consequently decreasing reflectance (Jaborek et al., 2018). It was published that more myoglobin can be found in meat of female animals (2.90 mg/g of meat) than in entire males (2.56 mg/g of meat) (de Araújo et al., 2017).

**TABLE 2:** Effect of *Romanov* lamb gender on chemical composition and quality parameters of *Gluteus medius* muscle

Parameter		Male lamb	Female lamb
Dry matter, %		28.95 ± 1.42	29.74 ± 1.06
Protein, %		17.92 ± 0.63	17.83 ± 0.81
Fat, %		9.99 ± 1.58	10.89 ± 1.15
Ash, %		1.04 ± 0.05	1.02 ± 0.04
Color coordinates	L*	40.21 ± 0.68^b^	39.00 ± 0.26^a^
	a*	20.99 ± 0.32^a^	22.74 ± 0.78^b^
	b*	5.78 ± 0.48^a^	8.50 ± 0.72^b^
pH after 24 h		5.80 ± 0.06^b^	5.66 ± 0.03^a^
pH after 48 h		5.63 ± 0.06^b^	5.48 ± 0.06^a^
Drip loss, %		1.26 ± 0.34^b^	0.69 ± 0.05^a^
Water holding capacity, %		61.70 ± 1.51	63.08 ± 0.40
Cooking loss, %		24.66 ± 2.58^b^	15.30 ± 1.66^a^
Shear force, kg/cm^2^		1.78 ± 0.10	1.44 ± 0.12

Note

Data expressed as means (*n* = 17) ± standard error.

^a,b^Mean values with different letters along the row are significantly different (*p* ≤ .05).

L*, lightness; a*, redness; b*, yellowness.

Lamb meat pH was significantly influenced by the gender factor, and, in all cases, lower pH was obtained for female meat (pH of male meat after 24 and 48 h was higher by 2.4% and 2.7%, respectively, compared to female lamb meat). An increase in the pH of meat can increase the activity of cytochrome oxidase by reducing the uptake of oxygen by myoglobin, which results in a purplish red color (Calnan et al., 2016). pH is the main meat quality parameter; it is very important commercially and has effects on meat texture, color, and water holding capacity (WHC), as well as sensory properties and overall acceptability of meat (Popp et al., 2015). High pH values of meat influence storage quality and can adversely affect flavor and aroma. Meat shelf life is reduced when pH exceeds 5.8, and as pH increases, meat becomes darker and affects consumer purchase decisions (Hopkins & Mortimer, 2014). A faster decrease in pH in females than in males can be explained by a higher concentration of glycogen in females due to lower sexual activity (de Lima et al., 2016).

Gender was not a significant factor in lamb meat WHC and tenderness; however, a significant influence of gender on meat cooking loss (CL) was found (38.0% higher CL for male lamb meat). In literature, the reports are inconsistent about the effect of gender on mentioned parameters. Some studies have shown that meat from male lambs was harder than that from the female lambs, whereas other reported no significant differences in meat quality traits between ewes and rams (Hopkins & Mortimer, 2014). Velasco et al. (2000) observed the impact of gender on WHC of *Talaverana* lamb meat. However, no gender effect on CL and tenderness of lamb meat was reported in that study. The differences in the CL and shear force (SF) between female and male lamb meat could be partly explained by the higher intramuscular fat content in female meat (Facciolongo et al., 2018).

The proximate chemical composition of the lamb meat is given in Table 2. It was observed that the chemical composition of meat was not affected by the lamb gender. The meat from male lambs contained 28.95% of DM, 17.92% of protein, 9.99% of fat, and 1.04% of ash. The meat from female lambs contained 29.74% of DM, 17.83% of protein, 10.89% of fat, and 1.02% of ash.

According to the literature, the chemical composition of lamb meat depends on breed, age, gender, genetic characteristics, and type of muscle fibers (de Lima et al., 2016). Similar results were obtained for *Romanov* crossbred muscle chemical composition by Kuchtík et al. (2012). However, the meat of other lamb breeds, such as *Altamurana* and *Corriedale*, has similar values for protein, ash, and DM, but differs in fat content, compared to *Romanov* crossbreds (della Malva et al., 2016; Jaworska et al., 2016). In another study, female lambs showed a higher percentage of carcass fat than males (Armero & Falagán, 2015). It was published that *Texel* variety male and female lambs have a significantly (*p* < .01) different muscle fat content adjusted to the same carcass weight (de Lima et al., 2016). No significant differences in intramuscular fat content between the goat breeds *Moxotó* and *Canindé* were found (Ablikim et al., 2016). Variations in meat fat content occur mainly due to changes in the balance between dietary energy and nutrient requirements (de Araújo et al., 2017).

### 3.3 Biogenic amine content in lamb meat

**FIGURE 1:**
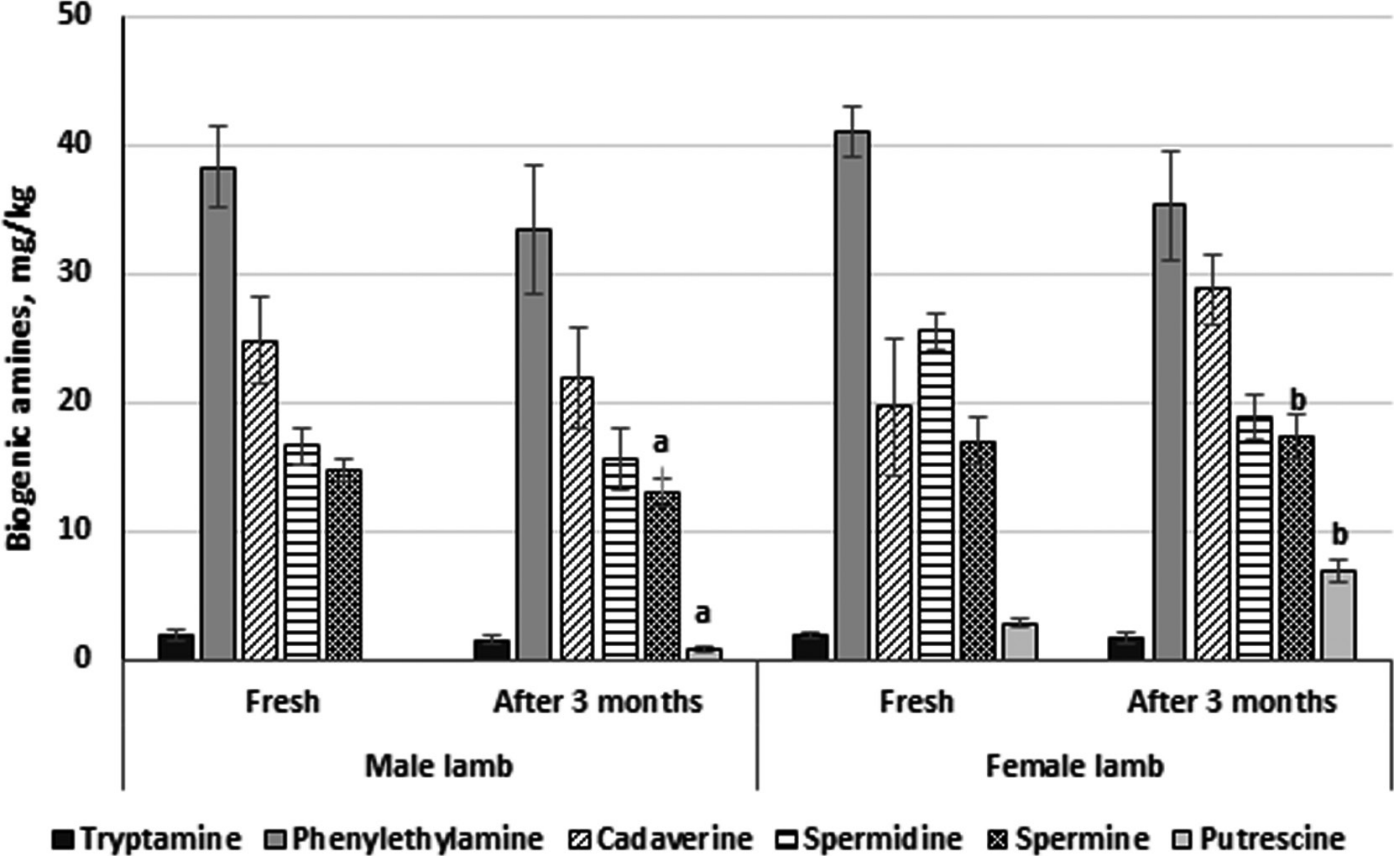
Biogenic amine content (mg/kg) of *Gluteus medius* muscle of *Romanov* lamb male and female meat (fresh and after 3 months of storage at −18°C). Data are represented as means (*n* = 17) ± standard error. ^a,b^Mean values denoted with different letters are significantly different (*p* ≤ .05)

The BA content in fresh lamb meat and after 3 months of storage is given in Figure 1. Gender was not a significant factor in BA concentration in lamb meat, except for spermine and putrescine after 3 months of storage (*p* ≤ .05). Histamine and tyramine were not detected in all analyzed samples. Putrescine was not found in fresh male samples, while its content in fresh female meat was 2.84 mg/kg. After storage, spermine and putrescine contents were significantly higher in female lamb meat compared to male meat (by 33.6% and 8.9‐fold, respectively). In lamb meat, BA concentration was found to be in the order tryptamine < putrescine < spermine <spermidine < cadaverine < phenylethylamine. BAs are generally formed by amination or transamination of aldehydes and ketones or decarboxylation of free amino acids (Jain et al., 2015). The content of BAs is highly affected by the presence of decarboxylase‐positive bacteria, meat composition (the content of free amino acids, pH, and additives), endogenous or microbial proteases, and handling and storage conditions (time/temperature, packaging, temperature abuse, etc.) (Triki et al., 2018). The most significant BAs in meat and meat products are tyramine, cadaverine, putrescine, and histamine (Jairath et al., 2015). In fresh meat, spermine and spermidine can be found naturally in significant amounts (20–60 mg/kg and less than 10 mg/kg, respectively) (Stadnik & Dolatowski, 2010). Similar levels of spermine were also observed in our study; however, the levels of spermidine were higher than 10 mg/kg. Usually, tyramine, cadaverine, and putrescine are formed during meat storage (Jairath et al., 2015). Coliform microflora and lactic acid bacteria produce tyramine, whereas *Enterobacteriaceae* and *Pseudomonas* are known to produce putrescine, cadaverine, and histamine (Hutařová et al., 2013). According to our results, only cadaverine was found in higher levels, while histamine and tyramine were not detected in all samples.

Meanwhile, phenylethylamine was the predominant BAs in all lamb meat. The determined level of this Bas is slightly higher than the set limit (30 mg/kg) (Triki et al., 2018). The higher content of phenylethylamine could be related with the presence of microflora with phenylalanine decarboxylation capacity or increased availability of free phenylalanine in lamb meat (Triki et al., 2018). However, a lower storage temperature minimizes BAs accumulation through inhibition of microbial growth and the reduction in enzyme activity. This tendency was also observed in most of the analyzed lamb meat samples of this study. It has been reported that in over 4 months’ storage at 1°C, only tyramine was detected consistently in beef carcasses among all the other BAs (histamine, phenylethylamine, tryptamine, and tyramine) (Jairath et al., 2015). The biogenic amines index (BAI), which is the sum of histamine, cadaverine, putrescine, and tyramine, is calculated to assess the quality of the meat (Wójcik et al., 2020). BAI for fresh meat does not exceed 5 mg/kg, for acceptable consumption – between 5 and 20 mg/kg, while the BAI range of 20–50 mg/kg shows poor hygiene quality. In our study, the following BAI values were found for male and female meat: 24.8 mg/kg and 22.66 mg/kg (fresh and stored, respectively); and 22.53 mg/kg and 35.69 mg/kg (fresh and stored, respectively). According to this, all male meat and fresh female meat showed initial signs of deterioration, whereas the quality of stored female meat was inferior to male.

### 3.4 Influence of lamb gender on lamb meat fatty acid composition

The FA composition of meat from lambs of different gender is given in Table 3. The significant effect of lamb gender was found on the levels of 10‐heptadecenoic (C17:1), linoleic (C18:2n – 6), and total polyunsaturated acids, as well as total *trans* isomers in analyzed lamb meat (*p* = .003; *p* = .018; *p* = .005, *p* = .005, respectively). The significantly higher levels of 10‐heptadecenoic (C17:1) and linoleic (C18:2n – 6) acids were found in the female lamb meat (by 72.5% and 16.0%, respectively) compared to male meat. Moreover, the level of polyunsaturated acids was higher by 10.7% in the female meat compared to male. However, the meat from female lambs contained the higher amount of total *trans* isomers (by 26%).

**TABLE 3:** Fatty acid composition (% of total fatty acids) of *Gluteus medius* muscle of *Romanov* lamb male and female meat

	Fatty acid	Male lamb	Female lamb
Saturated fatty acids	Capric C10:0	0.13 ± 0.02	0.12 ± 0.03
	Lauric C12:0	0.68 ± 0.09	0.53 ± 0.04
	Myristic C14:0	3.68 ± 1.15	3.54 ± 0.60
	Palmitic C16:0	25.47 ± 0.91	24.05 ± 0.58
	Stearic C18:0	22.42 ± 1.41	21.67 ± 1.22
	Arachidic C20:0	0.20 ± 0.02	0.20 ± 0.06
Monounsaturated fatty acids	Myristoleic C14:1	0.17 ± 0.02	0.19 ± 0.07
	Cis‐10‐pentadecenoic C15:1	0.86 ± 0.08	0.93 ± 0.02
	Palmitoleic C16:1n − 7	2.75 ± 0.07	2.92 ± 0.47
	10‐Heptadecenoic C17:1	0.47 ± 0.10^a^	1.71 ± 0.35^b^
	Oleic C18:1n − 9c	32.37 ± 1.45	31.94 ± 2.74
	Nervonic C24:1	1.16 ± 0.14	1.05 ± 0.10
Polyunsaturated fatty acids	Linoleic C18:2n − 6	2.53 ± 0.11^a^	3.01 ± 0.08^b^
	gamma‐Linolenic C18:3n − 6	2.60 ± 0.05	2.75 ± 0.09
	alpha‐Linolenic C18:3n − 3	1.50 ± 0.03	1.69 ± 0.07
	cis‐8,11,14‐Eicosatrienoic C20:3n − 6	0.05 ± 0.01	0.06 ± 0.02
	Arachidonic C20:4n − 6	0.02 ± 0.01	0.03 ± 0.01
	EPA C20:5n − 3	0.11 ± 0.02	0.11 ± 0.03
	DHA C22:6n − 3	0.18 ± 0.03	0.18 ± 0.02
*trans* isomers	Elaidic C18:1n − 9 t*rans*	2.65 ± 0.44^a^	3.34 ± 0.11^b^
	Linolelaidic C18:2n − 6 *trans*	0.01 ± 0.00	0.01 ± 0.00
Total saturated		52.58 ± 1.57	50.10 ± 1.41
Total monounsaturated		37.78 ± 1.25	38.73 ± 1.36
Total polyunsaturated		6.98 ± 0.21^a^	7.82 ± 0.14^b^
Total *trans* isomers		2.66 ± 0.02^a^	3.35 ± 0.04^b^

Note

Data expressed as means (*n* = 17) ± standard error.

^a,b^Mean values with different letters along the row are significantly different (*p* ≤ .05).

The FA composition has a significant influence on the sensory attributes of meat and its nutritional value (Wood & Enser, 2017). The data about FA composition in lamb meat are varied. Camacho et al. (2017) reported that *Canarian Hair* female lambs had a higher content of monounsaturated FA (mainly oleic acid) than males. Erasmus et al. (2017) found that females had more α‐linolenic acid (C18:3n – 3) and polyunsaturated FA in their subcutaneous fat than male lambs. In our study, a higher content of polyunsaturated FA was also found in female meat, while the content of C18:3n – 3 was similar for both genders. Moreover, the small amount of *trans* FA was found in female and male meat. *Trans* FA naturally occur in ruminant meat due to anaerobic bacteria fermentation in rumen (Lichtenstein, 2016).

### 3.5 Cholesterol concentration in lamb meat and malondialdehyde changes during lamb meat storage

**FIGURE 2:**
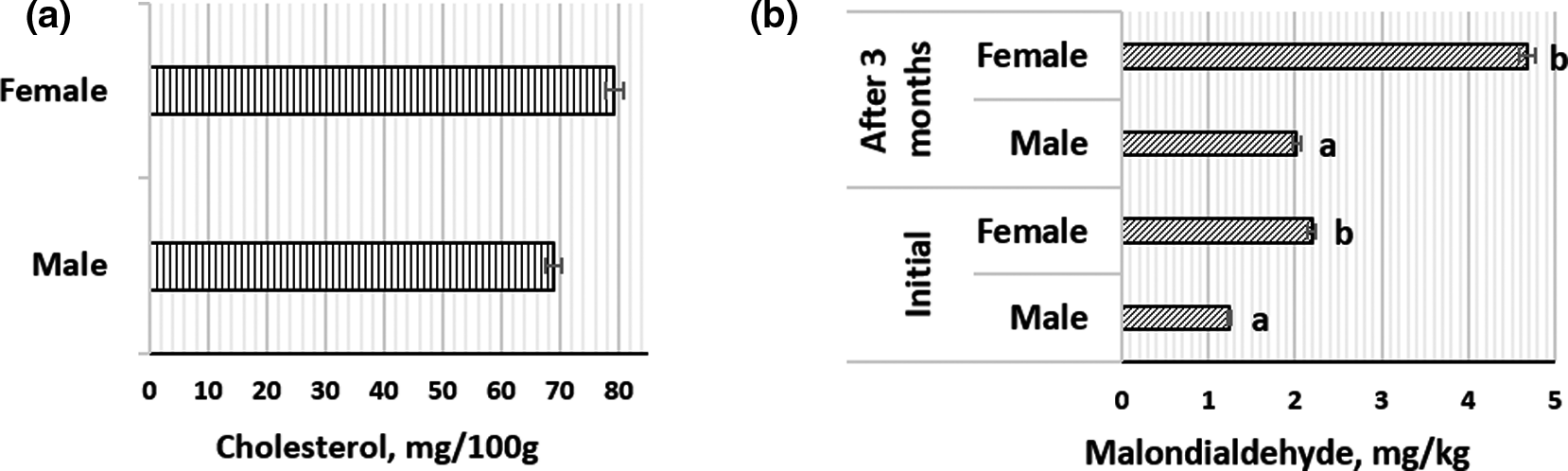
Cholesterol content (mg/100 g) of *Gluteus medius* muscle of *Romanov* lamb male and female meat (a). Malondialdehyde (MDA) (mg/kg) content of *Gluteus medius* muscle of *Romanov* lamb male and female meat (fresh and after 3 months of storage at −18°C) (b). Data are represented as means (*n* = 17) ± standard error. ^a,b^Mean values denoted with different letters are significantly different (*p* ≤ .05)

Cholesterol concentrations of 68.99 ± 5.42 mg/100 g and 79.85 ± 5.93 mg/100 g were found in male and female lamb meat, respectively (Figure 2a). Gender was not a significant factor in cholesterol concentration in lamb meat; however, gender had a significant influence on the malondialdehyde (MDA) concentration (in fresh meat and after 3 months of storage, *p* = .05) (Figure 2b). In all cases (in fresh meat and after 3 months of storage), a higher concentration of MDA was found in female lamb meat (by 43.4% and 56.8%, respectively).

The cholesterol results are in agreement with the findings of other authors. In the study of Yousefi et al. (2018), cholesterol content in *Chall* lamb meat varied from 47.5 to 52.9 mg/100 g, and no influence of genotype was obtained. Dávila‐Ramírez et al. (2018) reported cholesterol values of 50.4–63 mg/100 g in *40 Dorper* × Pelibuey crossbred male lamb meat. The MDA content indicates lipid peroxidation in food products and can be attributed to the fat and polyunsaturated FA content in intramuscular depots (Jung et al., 2016). This secondary product can influence undesirable changes in the sensory properties of the products and a decrease in the nutritional value of foods. In the literature, findings about MDA in lamb meat are inconsistent. Nudda et al. (2013) reported a higher MDA content in *Sarda* female lambs than males. These results are in agreement with those obtained in our study. The increased content of MDA in female meat could be related with the higher content of polyunsaturated FA. However, de Araújo et al. (2017) did not find a significant effect of gender on MDA content in *Morada Nova* lamb meat.

## 4 CONCLUSIONS

The *Romanov* lamb breed is characterized by a high productive performance and is frequently crossed with other lamb breeds. However, there is little or no information about how the gender could influence the carcass traits and quality parameters of *Romanov* lamb meat. The outcome of this study showed that *Romanov* lamb gender had a significant influence on several carcass parameters (sternum/breastbone, ribs, right shoulder, and bones (left and right) of the rear foot). Male meat had a significantly higher lightness, lower redness, and higher values of pH and cooking loss. The content of 10‐heptadecenoic (C17:1), linoleic (C18:2n – 6), total polyunsaturated FA, and total *trans* isomers were higher by 72.5%, 16.0%, 10.7%, and 26%, respectively, in female lamb meat, compared to male meat. Increased MDA content in female meat could be related with the higher content of polyunsaturated FA. However, gender was not a significant factor in lamb meat proximate composition, cholesterol concentration, or the content of most BAs. Finally, the results obtained are important for lamb breeders, as well as for producers and consumers, and supplement the scarce database about the parameters of *Romanov* breed meat of different gender. According to these results, further preparation of lamb meat can be planned in order to prepare the highest quality and acceptability of meat products for consumers.

## CONFLICT OF INTEREST

The authors declare that they do not have any conflict of interest.

## AUTHOR CONTRIBUTION

**Dovile Klupsaite:** formalAnalysis (equal) ; investigation (equal); writingOriginalDraft (equal); writingReviewEditing (equal). **Vilija Buckiuniene:** conceptualization (equal) ; methodology (equal); resources (equal); writingOriginalDraft (equal). **Saulius Bliznikas:** formalAnalysis (equal) ; investigation (equal). **Sonata Sidlauskiene:** formalAnalysis (equal) ; investigation (equal). **Agila Dauksiene:** dataCuration (equal) ; validation (equal); writingReviewEditing (equal). **Jolita Klementaviciute:** formalAnalysis (equal) ; investigation (equal); writingOriginalDraft (equal). **Andrius Jurkevicius:** dataCuration (equal) ; formalAnalysis (equal); investigation (equal). **Gintare Zaborskiene:** dataCuration (equal) ; validation (equal); writingReviewEditing (equal). **Elena Bartkiene:** conceptualization (equal) ; formalAnalysis (equal); methodology (equal); supervision (equal); writingOriginalDraft (equal); writingReviewEditing (equal).

## ETHICAL APPROVAL

The animals were cared for in accordance with the Lithuanian State Food and Veterinary Service requirements (“Requirements for the Keeping, Maintenance and Use of Animals Intended for Science and Education Purposes,” approved by the order of the Lithuanian Director of the State Food and Veterinary Service, 31/10/2012, No. B1‐866).
